# Biomimetic phototherapy in cancer treatment: from synthesis to application

**DOI:** 10.1080/10717544.2021.1983082

**Published:** 2021-10-01

**Authors:** Yifan Zhao, Cuixia Shi, Jie Cao

**Affiliations:** a Department of Pharmaceutics, School of Pharmacy, Qingdao University, Qingdao, PR China;; b Department of Gynecology and Obstetrics, The People’s Hospital of Feixian, Linyi, PR China

**Keywords:** Biomimetic camouflage, photodynamic therapy, photothermal therapy, cell membrane, biomimetic enzyme

## Abstract

Phototherapy, with minimally invasive and cosmetic effect, has received considerable attention and been widely studied in cancer treatment, especially in biomaterials field. However, most nanomaterials applied for the delivery of phototherapy agents are usually recognized by the immune system or cleared by liver and kidney, thus hindering their clinical applications. To overcome these limitations, bionic technology stands out by virtue of its low antigenicity and targeting properties, including membrane bionics and bionic enzymes. In this review, we will summarize the up-to-date progress in the development of biomimetic camouflage-based nanomaterials for phototherapy, from synthesis to application, and their future in cancer treatment.

## 1. Introduction

Cancer has been one of the worldwide health problems for centuries, accompanied by the increasing trend of young patients (Miller et al., 2020; Siegel et al., 2020). Although chemotherapy, radiotherapy, and surgery are primary approaches for cancer treatment, their low selectivity and severe side effects always results in tumor recurrence and metastasis. Therefore, searching for an effective oncotherapy method has been a constant focus for many decades. Phototherapy, including photothermal therapy (PTT) and photodynamic therapy (PDT) (Cai et al., 2018), with minimally invasive, low cumulative toxicity, and cosmetic effect, has emerged as a new paradigm toward precise cancer therapy (Zhu et al., 2018). Harnessing the absorption of light to achieve the therapeutic response is the central concept of phototherapy. PDT is based on molecular oxygen, photosensitizer (PS), and irradiated light to exert an anti-tumor effect. Typically, PDT involves the generation of cytotoxic reactive oxygen species (ROS), such as singlet oxygen (^1^O_2_), under specific wavelengths of light to kill cancer cells (Chilakamarthi & Giribabu, 2017; Alzeibak et al., 2021). PTT, as is implied by the name, under the irradiation of the laser, a photothermal conversion agent can convert light energy into thermal energy, which can effectively kill cancer cells (Wang et al., 2019a,b).

Although phototherapy has many advantages in cancer treatment, single PSs, or photothermal agents are exogenous to the human body and lack of targeting properties, which make it difficult for PDT or PTT to target diseased tissues alone (Peng et al., 2018; Huang et al., 2019; Riera-Domingo et al., 2020). Therefore, researchers developed various nanomaterials to deliver the phototherapeutic agents that can improve the pharmacokinetic properties, and tumor-targeting ability of the agents (Yu et al., 2019; Chen et al., 2019a,b; Yu et al., 2020). However, most nanomaterials, once injected into the body, are usually recognized by the immune system as foreign substances that produce an immune response. In addition, the clearance of nanoparticles (NPs) by liver and kidney seriously restricts the clinical application of nanomaterials (Chou et al., 2011; Dobrovolskaia et al., 2014). In comparison, bionic technology stands out by virtue of its low antigenicity and targeting properties.

**Figure 1.**
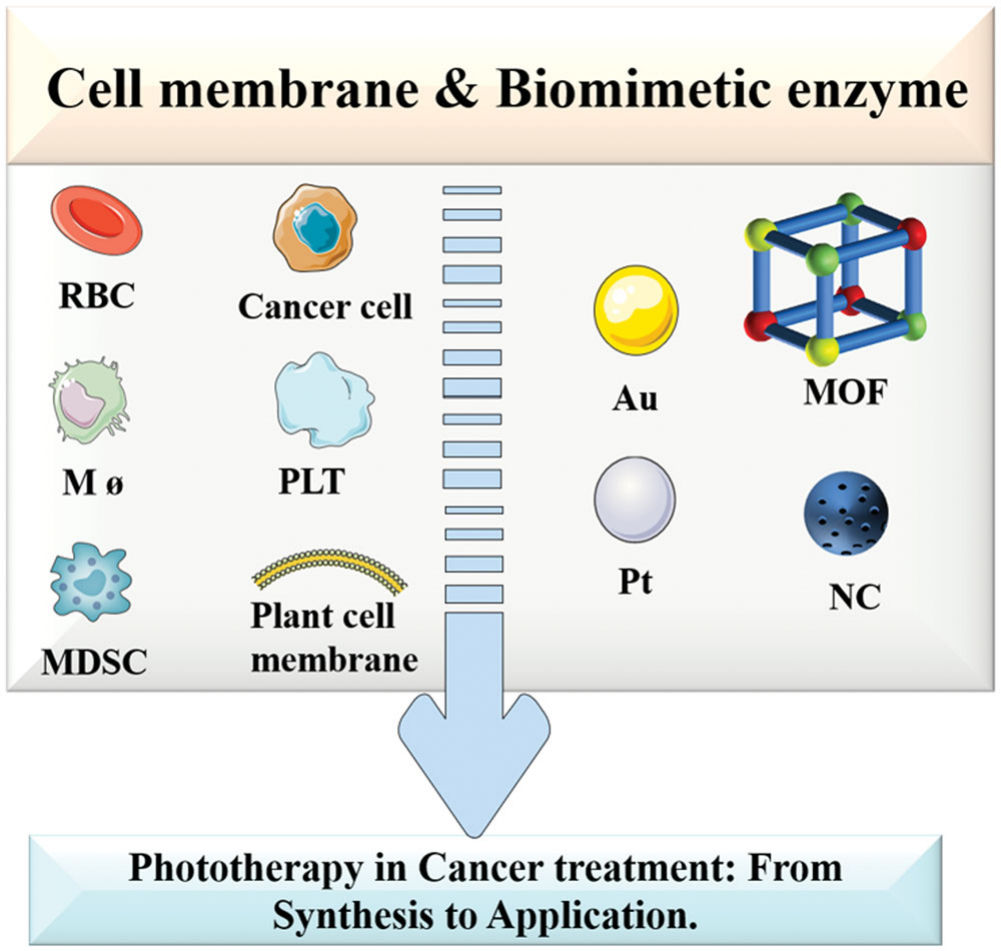
Overview of the biomimetic camouflage-based phototherapy for cancer treatment.

Based on this, biomimetic modification of nanomaterials becomes a good choice. For example, various cell membranes, including red blood cell (RBC) membrane (Liu et al., 2018a,b), cancer cell membrane (Sun et al., 2020), platelet membrane (PLTM) (Wei et al., 2018), myeloid-derived suppressor cell (MDSC) membrane (Yu et al., 2018), macrophage membrane (Liu et al., 2021), and plant cell membrane (Ouyang et al., 2018) are camouflaged to encapsulate NPs (Zhao et al., 2020), hydrogels (Yuan et al., 2020), nanoemulsion (Zhang et al., 2021), and so on, combining with phototherapy to improve tumor targeting and drug release. In addition, the emergence of biomimetic enzymes (such as metal enzymes (Zhang et al., 2020a,b), metal-organic framework (MOF) (Wang et al., 2019a,b), N-doped carbon (Xu et al., 2020) has greatly improved the shortcomings of natural enzymes. In previous reviews, researchers have summarized biomimetic camouflage-based nanomaterials for cancer diagnosis and therapy, including the types of cell membranes and the application of cell membrane bionic technology. But few people summarize the application of biomimetic camouflage-based nanomaterials and biomimetic enzyme in phototherapy, especially from the synthesis strategies to application. Therefore, a comprehensive and in-depth depiction of the whole scene of the recent advances of biomimetic camouflage-based phototherapy is desirable. Therefore, in this review, we summarize the up-to-date progress in the development of biomimetic camouflage-based nanomaterials for phototherapy, from synthesis to application, as well as the future development in cancer treatment (Figure 1).

## 2. Cell-membrane coating technology

For cell membrane coated nanomaterials, the cell membrane is usually wrapped by physical co-extrusion; membrane extraction, preparation, and fusion of core nanomaterials are the most common steps. For the extraction of nucleated cells, such as RBCs, the cell membrane is obtained by hypotonic treatment, freeze-thaw, or ultrasonic destruction, and the suitable nanomaterials are extruded through the porous polycarbonate membrane. For different cell membranes, membranes are firstly extracted from different cells and then fused together by ultrasound. The fusion film was coated on the nanomaterials and fused with ultrasound.

However, there are still some challenges in the preparation, such as low yield, poor repeatability, and so on. To overcome these obstacles, researchers pioneered other preparation approaches. For example, Rao et al. (2018) used microfluidic electroporation technology to realize the preparation of large quantities of biomimetic NPs wrapped in cell membrane. Briefly, this electroporation microfluidic system consists of five parts, namely two entrances, a Y-shaped channel, an S-shaped channel, an electroporated area and an outlet. The prepared NPs and cell membrane-derived vesicles were injected into the microfluidic chip, and after fully mixing in the channel, the NPs entered the vesicles under the action of electric pulse. Finally, the NPs camouflaged by the cell membrane were obtained.

## 3. Cell membrane biomimetic platform for phototherapy

Biomimetic membrane camouflage, uses natural cell membranes, could simulate the important role of various cellular components in the physiological and pathological process. For the past decades, researchers have focused on developing biomimetic nanomaterials for drug delivery. These biomimetic NPs with natural cell membrane exhibit some unique advantages, such as good biocompatibility, low immunogenicity, reducing the nonspecific uptake of NPs by the reticuloendothelial system, and prolonging the circulation time of NPs in the blood. Notably, the surface modification of the cell membrane can endow NPs with the ability to actively target diseased tissues. So far, enormous efforts have been devoted to applying membrane materials for delivering phototherapy agents for enhanced anti-tumor treatment, such as RBC membrane (Pei et al., 2018; Liu et al., 2018a,b; Shi et al., 2020), cancer cell membrane (Li et al., 2017; Wang et al., 2020a,b), PLTM (Ma et al., 2021), macrophage membrane (Liu et al., 2021), phagocyte membrane (Hu et al., 2020), MDSC membrane (Zhang et al., 2020a,b), and fused membranes (Xu et al., 2021).

### 3.1. RBC

RBCs, as is well known, are the most abundant and longest-lived blood cells in the blood (Foller et al., 2008). Studies have shown that based on the ‘don't eat me’ signal from the immune protein CD47 on the erythrocyte membrane to macrophages (Fang et al., 2017; Xia et al., 2019), the average survival life of RBCs in adults is as high as 120 d (Kroll et al., 2017; Wibroe et al., 2017), allowing RBCs to deliver oxygen to body tissues for a long time. Therefore, due to the long-term blood circulation, excellent biocompatibility, and low immunogenicity, RBC-coated nanomaterials show broad application potential in tumor diagnosis and treatment. Especially, RBC-based phototherapy showed great prospects in effective bionic antitumor strategy (Gao et al., 2013; Liu et al., 2018a,b; Xia et al., 2019).

**Figure 2.**
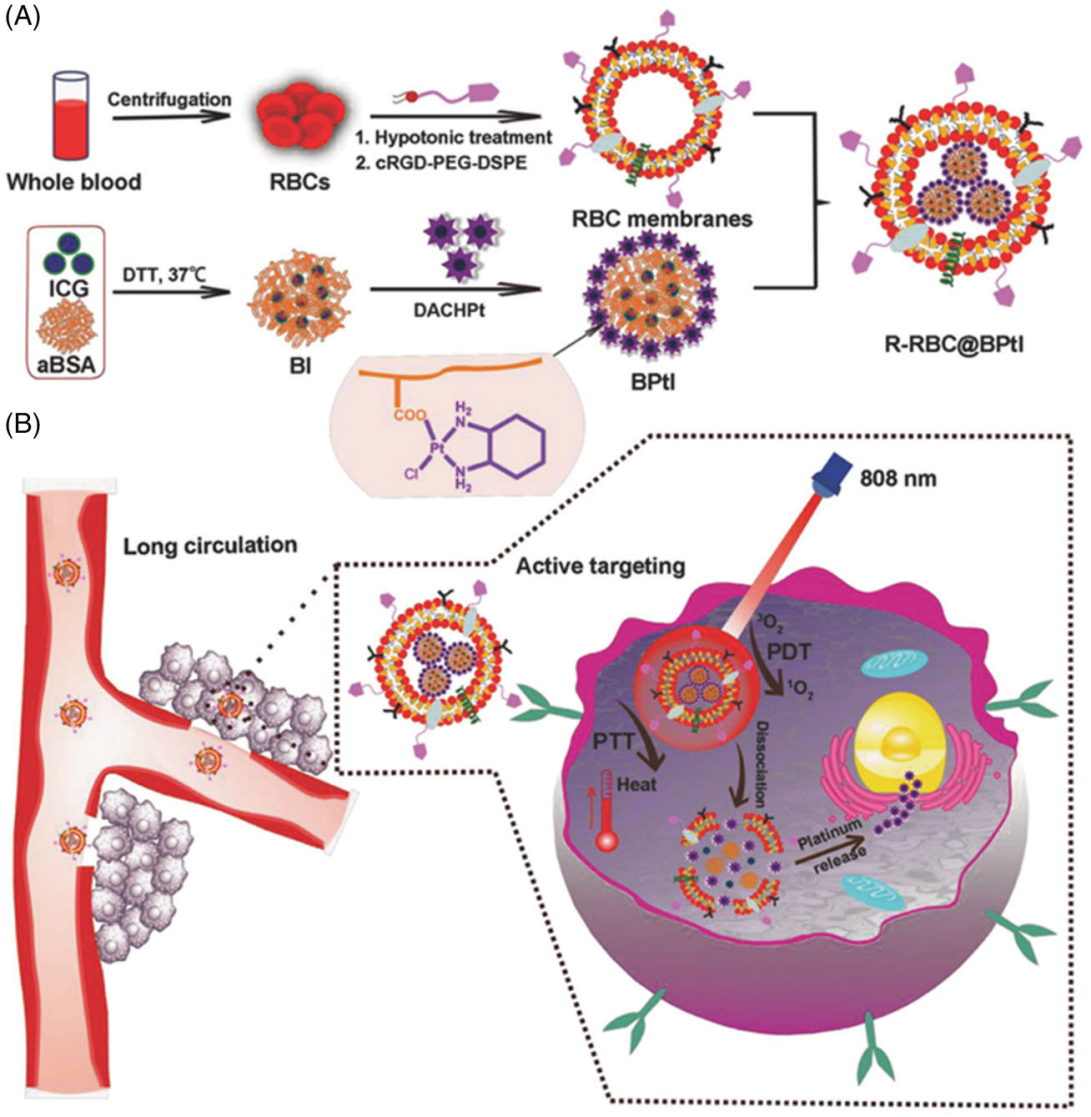
(A) Schematic illustration of the design of light‐activatable biomimetic nanoerythrocytes by a process involving RBC membrane‐cloaking of two‐in‐one nanoparticle coloaded photosensitizers (PS) and cis‐platinum (II). (B) R‐RBC@BPtI as a biomimetic combination therapeutic nanoplatform for tumor‐targeted and light‐triggered chemo‐phototherapy *in vivo*. Copyright 2018, John Wiley and Sons (Liu et al., 2018a).

RBC-based phototherapy showed great prospects in effective bionic antitumor strategy. Unfortunately, RBC lacks tumor targeting, which leads to insufficient accumulation and may be toxic to other normal cells, so many researchers developed ligand modified strategy or using hybrid membrane to improve tumor targeting delivery. Based on this, Liu et al. (2018a,b) designed and developed a light-triggered biomimetic nanoerythrocyte for tumor-targeted lung metastatic combined with phototherapy and chemotherapy (R-RBC@BPtI) (Figure 2). They first modified the erythrocyte membrane to own tumor targeting ability and then squeezed protein composite NPs loaded with photosensitive reagent (indocyanine green, ICG) and cis-platinum (II) (1,2-diaminocyclohexane-platinum (II), (DACHPt)) into the RBC membrane. Because of the effective coverage of the erythrocyte membrane, R-RBC@BPtI showed excellent immune escape and tumor-specific targeting ability, which significantly prolonged the circulation lifetime and high tumor accumulation. Under the laser irradiation of 808 nm, ICG produces heat energy to damage cells, and at the same time produces singlet oxygen, which plays a cytotoxic role. *In vivo* study demonstrated that the combined therapy of R-RBC@BPtI showed enhanced anti-tumor effect and low systemic toxicity. Subsequently, the researchers established a metastatic melanoma model to observe the inhibitory effect of R-RBC@BPtI on lung metastasis of B16F10 tumor. The lung metastases of mice in RBC@BPtI + laser group showed better anti-metastasis ability than other groups, illustrating the biomimetic nanoerythrocyte could be group an effective comprehensive treatment strategy for powerful ablation and inhibition of tumor metastasis.

Immunotherapy has already been shown to hold great promise in cancer treatments. Unfortunately, immunity does not work for all tumors, especially for ‘cold’ tumors (immune exhaustion). Therefore, how to comprehensively suppress the immunosuppressive tumor microenvironment (TME) is still a major challenge for immunotherapy to maximize the benefit. Phototherapy has been shown to induce immunogenic cell death (ICD), recruitment antigen-presenting cells to phagocytize tumor cell antigens and further activate T cell adaptive immune response (Li et al., 2019; Yang et al., 2020a,b,c; Alzeibak et al., 2021). Therefore, the particles coated with erythrocyte membrane can not only improve the effect of phototherapy but also improve the immune effect.

**Figure 3.**
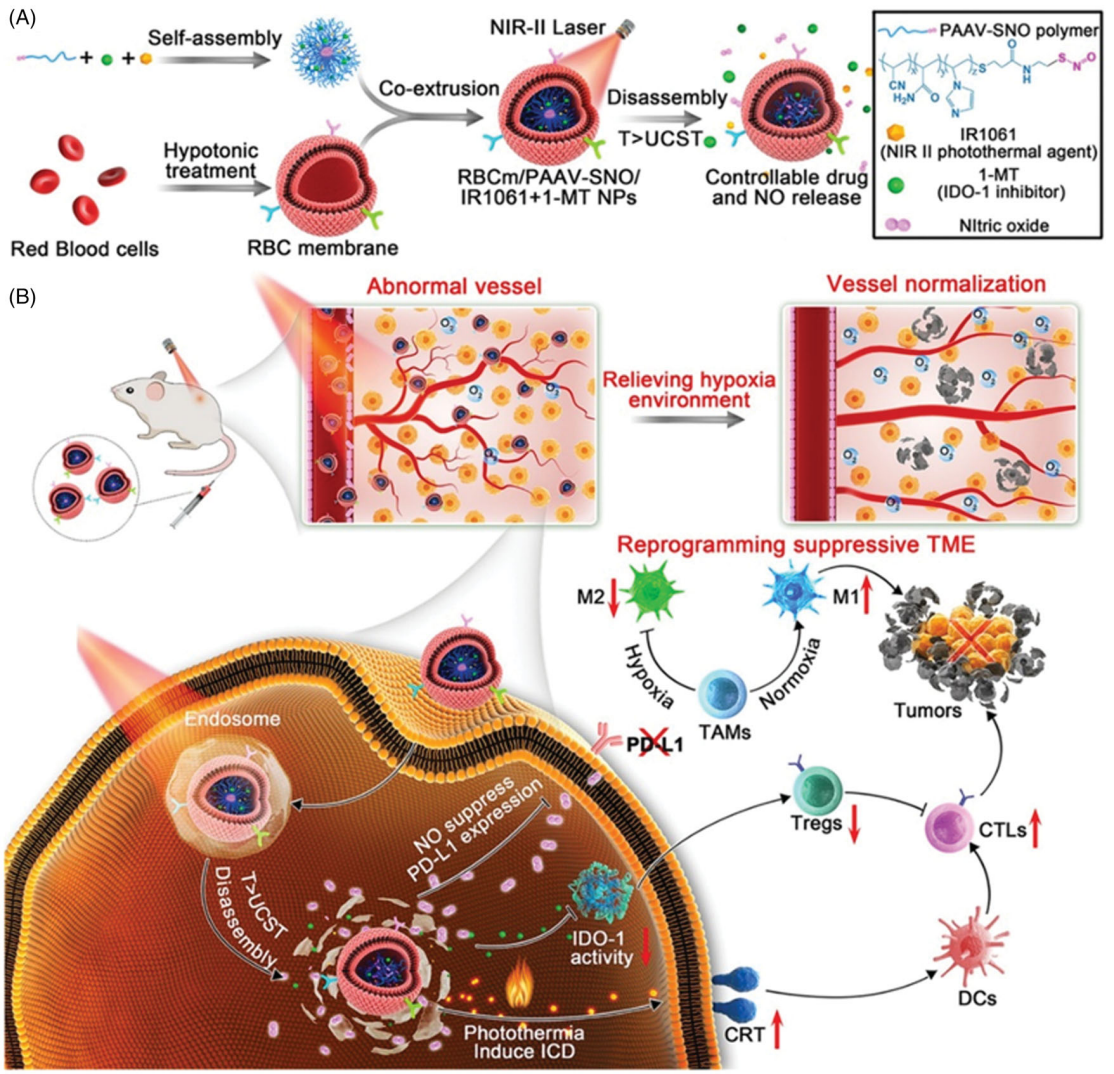
Schematic showing the structure and therapeutics releasing process of erythrocyte membrane-camouflaged nanobullets and (B) their capacities of reprogramming tumor immunosuppressive microenvironment and fighting immune cold tumor. Copyright 2020, American Chemical Society (Yang et al., 2020c).

Yang et al. (2020a,b,c) proposed a strategy that can not only increase tumor infiltration for lymphocyte recruitment but also comprehensively reprogram the immunosuppressive TME for enhanced phototherapy and immunotherapy (Figure 3). They synthesized a thermal-sensitive SNO donor-pendant copolymer (poly(acrylamide-co-acrylonitrile-co-vinylimidazole)-SNO, PAAV-SNO) and self-assembled it with NIR II photothermal agent IR1061 and indoleamine 2,3-dioxygenase 1 (IDO-1) inhibitor 1-methyl-tryptophan (1-MT). Subsequently, the erythrocyte membrane was wrapped around the self-assembly system to obtain RBCm/PAAV-SNO/1-MT + IR1061 NPs. Indoleamine 2,3-dioxygenase (IDO), a heme-containing oxidoreductase highly expressed in various neoplastic cells and APCs, can suppress the differentiation and function of effector T cells and promote the production of regulatory T cells, seriously affecting the immune response (Zhang et al., 2019; Wachowska et al., 2020; Zhao et al., 2020). The multi-functional nano-bullet has a long circulation feature in the body and high accumulation in the tumor site. Synchronously, it can be controlled drug release through NIR II to improve biological safety and avoid nonspecific drug leakage. More importantly, PTT can induce ICD to recruit the CD8^+^ cytotoxic T lymphocytes and activate the immune system to enhance the anti-tumor effect. In addition, 1-MT interferes with IDO-1 activity and generates NO in situ to normalize tumor vessels, resulting in the reprogramming of immunosuppressive TME. *In vivo* results showed that RBCm/PAAV-SNO/1-MT + IR1061 NPs could successfully restrict primary tumor growth and inhibit the pulmonary metastasis without obvious side effects, implying such multi-function nanoblastic can effectively reprogram the inhibitory TME and treat ‘immune cold’ tumors.

### 3.2. Cancer cell membrane

Despite the notoriety of cancer cells, cancer cells have their own unique and excellent characteristics, such as the unlimited proliferative potential and the ability to resist apoptosis. The biomarkers on the surface of the cancer cell membrane, such as T antigen-Galectin-3 and EpCAM, make cancer cells have homologous targeting and immunogenicity (Hainaut & Plymoth, 2013; Li et al., 2017; Tian et al., 2017), which could overcome the environment of immune clearance and nonspecific attachment *in vi*vo. Specially, the adhesion proteins on the surface of cancer cell types can mediate their effective self-recognition, allowing them to relocate to homologous tumor sites, and can effectively target cancer cells even in the presence of other heterogeneous tumor cells. Thus, surface modification of NPs by cancer cell membrane has attracted the attention of researchers for enhanced phototherapy. In light of the above considerations, surface modification of NPs by cancer cell membrane has attracted the attention of researchers for enhanced phototherapy.

**Figure 4.**
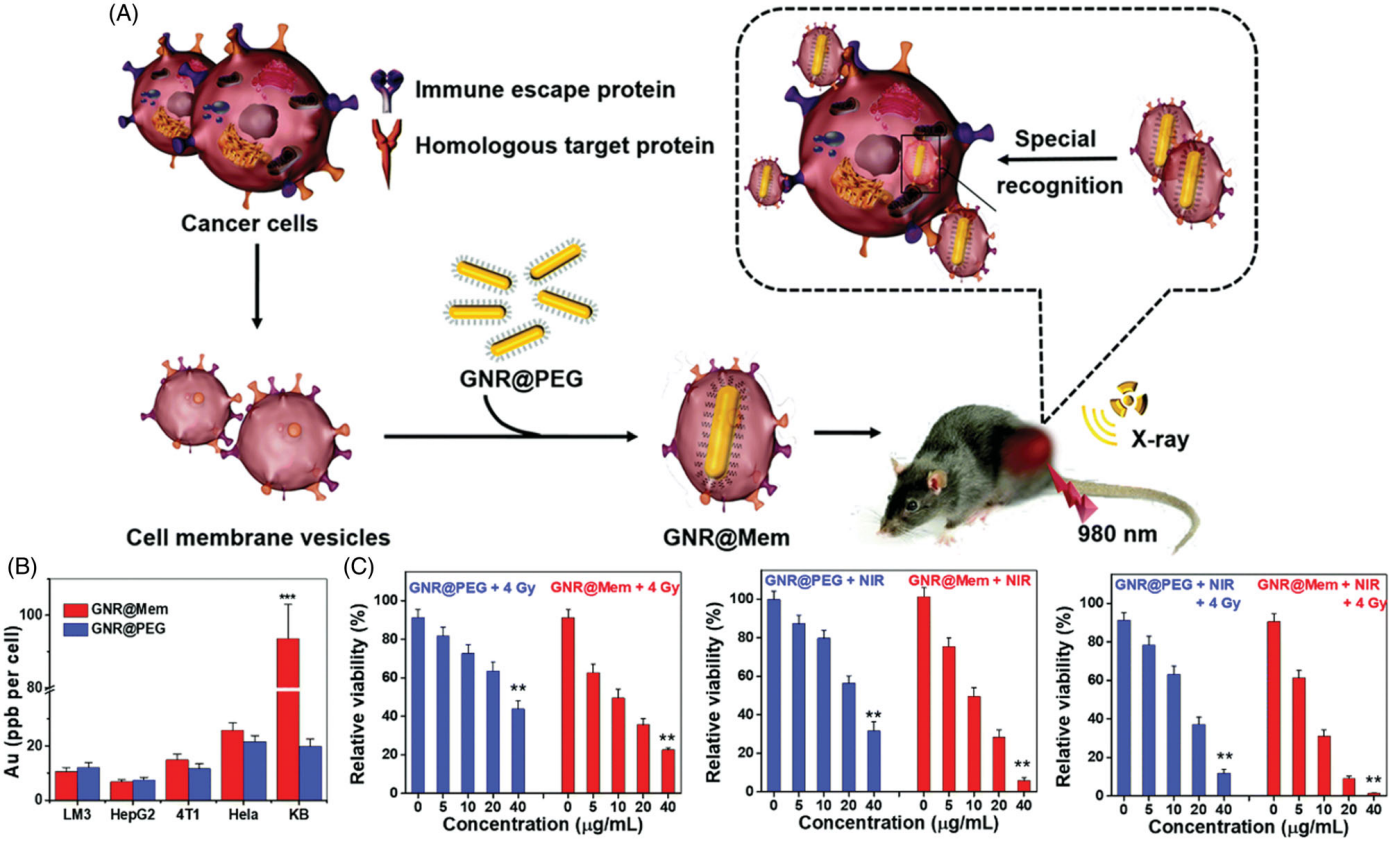
(A) Schematic illustration of the preparation of the cell membrane-coated gold nanorods, their accumulation in tumor tissues and cells, and their application in tumor photothermal therapy and radiotherapy. (B) Gold concentration in different cells treated with GNR@PEG or GNR@Mem for 24 h. Relative viability of KB cells incubated with various concentrations of GNR@PEG or GNR@Mem for 24 h, and then treated with C) X-rays (4 Gy), 980 nm NIR light, and X-rays + NIR light. Copyright 2020, Royal Society of Chemistry (Sun et al., 2020).

In the work of Sun et al. (2020), they fabricated a gold nanorod coated with the plasma membrane of oral squamous KB cancer cells (GNR@Mem) to augment the antitumor efficacy mediated by PTT and radiotherapy (Figure 4(A)). The targeting ability of GNR@Mem to different cells including LM3, HepG2, 4T1, HeLa, and KB cells were investigated. As illustrated in Figure 4(B), the higher uptake efficiency in KB cells of GNR@Mem among these cells confirmed the homotypic binding ability of GNR@Mem. Furthermore, under NIR light and X-rays, GNR@Mem presented a higher cytotoxicity in comparison with that of PEGylated control nanorod, resulting in enhanced photothermal and radiotherapy of GNR@Mem (Figure 4(C)). The researchers further studied the combined therapeutic effect of GNR@Mem on KB cell-generated tumor. The tumors in the control group(I) and NIR light group (II) showed rapid growth while tumors in GNR@Mem + NIR + X-ray group(VII) were completely destroyed. The same results were confirmed by H&E staining and Ki-67 staining. The results demonstrate that GNR@Mem is an excellent sensitizer for high-efficiency radiotherapy and PTT.

In addition, cancer cell membrane-based phototherapy can also be used in combination with chemotherapy or immunotherapy. For example, Zhao et al. (2020) combined the decitabine (DCT, DNA methylation inhibitors) and photothermal agent ICG and homologous targeting cancer cell membrane to form the nanomaterial BNP. First, the cell membrane of breast cancer was extracted by hypotonic lysis, ultrasound, and differential centrifugation. To obtain BNP, cell membrane vehicles were fused onto the polymeric core by co-extruding vehicles and polymeric core. After intravenous injection, BNP can be effectively internalized by cells due to homologous targeting.

**Figure 5.**
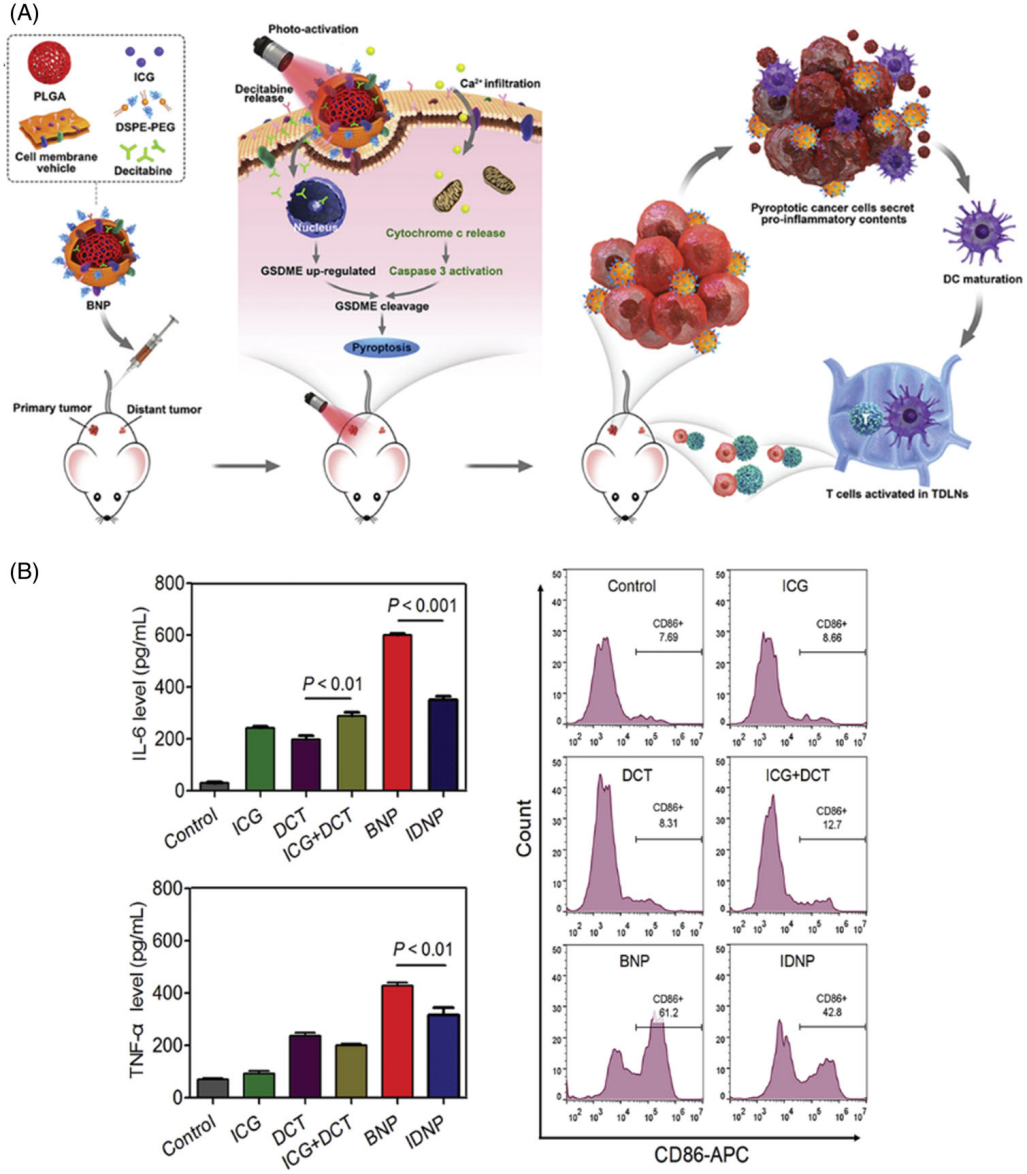
(A) The mechanism of photo-activated cell pyroptosis for solid tumor immunotherapy. (B) In vitro BMDCs maturation induced by the photo-activated cancer cell pyroptosis. Copyright 2020, Elsevier (Zhao et al., 2020).

After photoactivation, it can induce cell membrane penetration and increase intracellular Ca^2+^ concentration, thus promoting cytochrome *c* (cyt *c*) level and caspase-3 activity. Synchronously, DCT up-regulates the expression of gasdermin E (GSDME, the specific pyroptosis-inducing substrate cleaved by caspase-3) by inhibiting DNA methylation, which leads to the cleavage of caspase-3 and enhanced cancer cell pyroptosis (Boise and Collins, 2001; Shi et al., 2017; Kesavardhana et al., 2020). Cell pyroptosis, as a highly inflammatory programmed cell death, can alleviate immunosuppression and promote systemic immune response in solid tumors. Therefore, as shown in Figure 5, when BNP was co-cultured with cancer cells, IL-6 and TNF-α secreted by BMDCs (mouse bone marrow-derived dendritic cells [DCs]) were remarkably increased compared with other groups. Synchronously, the expression levels of CD86 and CD11c were also significantly increased. Furthermore, immunofluorescence staining showed that the infiltrating CD8^+^T cells and CD4^+^T cells in the BNP group were significantly higher than those in other treatment groups. These results prove that BNP-mediated cell pyroptosis activated anti-tumor immunity and showed an efficient inhibitory effect on tumor growth. Hence, biomimetic cancer cell membrane–camouflaged NPs for cancer phototherapy open up a window for tumor immunotherapy.

### 3.3. Platelet membrane

Platelets (PLTs) are small bioactive cytoplasms that fall off from the cleavage of mature megakaryocyte cytoplasm in bone marrow, which can respond to vascular injury and invasive microorganisms. On the other hand, it is demonstrated that PLTs can cover circulation tumor cells (CTC) to protect tumor cells from immune elimination and spread to new tissues (Hu et al., 2015; Sim et al., 2016). CD47 immunomodulatory protein is also present on the surface of the PLTM. The activated PLTM protein P-selectin is highly expressed and can specifically adhere to CD44 receptors on the surface of cancer cells (Gay & Felding-Habermann, 2011). Based on the excellent properties of PLT, the biomimetic camouflage drug delivery system of PLTM shows broad application potential for specific adhesion and removal of pathogens of damaged vascular adhesion, as well as a targeted attack on tumor sites.

**Figure 6.**
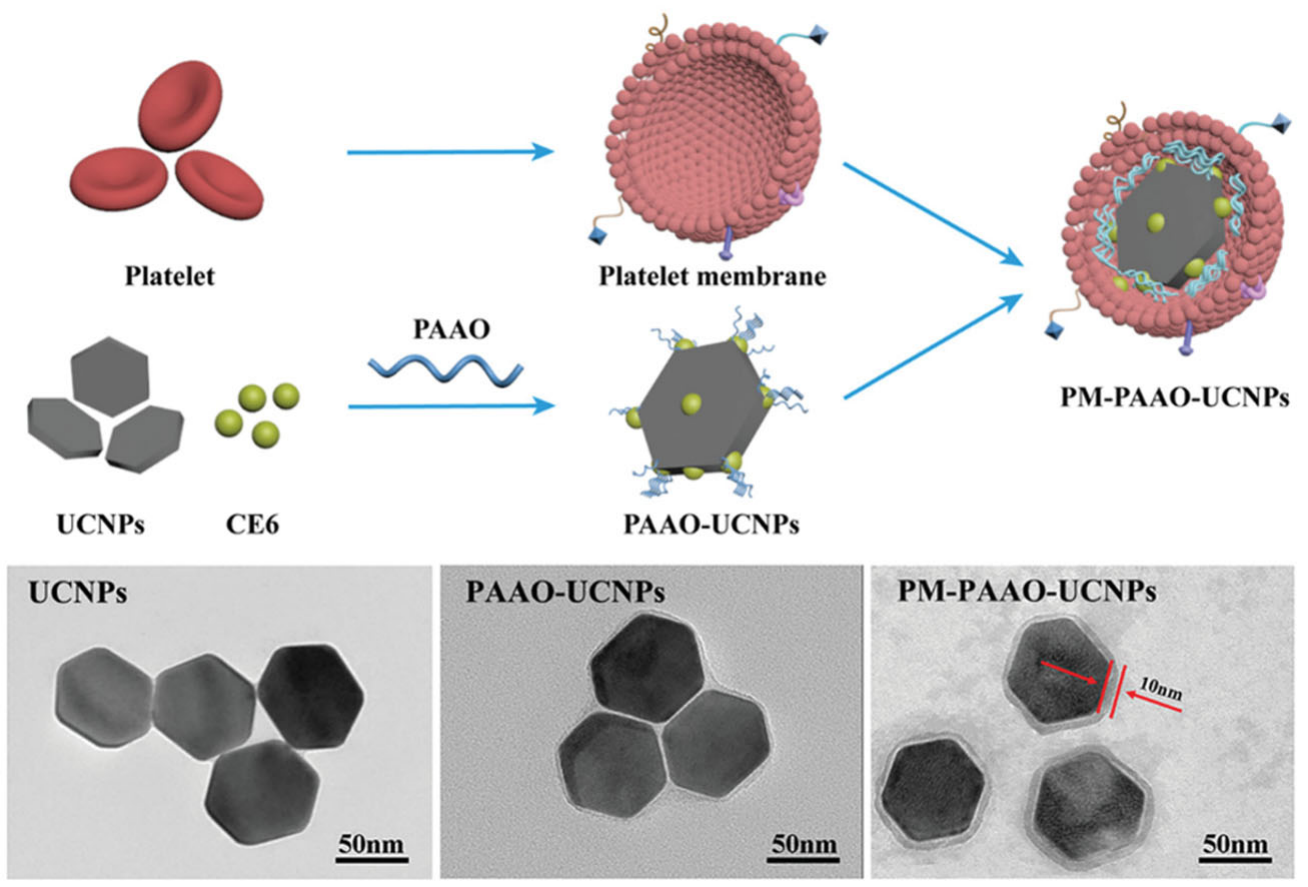
Design and characterization of PLTM‐PAAO‐UCNPs. Copyright 2021, John Wiley and Sons (Ma et al., 2021).

Inspired by the ability of PLTM, researchers have paid attention to PLTM-based phototherapy (Wei et al., 2018; Xu et al., 2018; Chen et al., 2019a,b; Ma et al., 2021). For example, Ma et al. (2021) designed a PLT-mimic system in which the bleeding PLTM encapsulated upconversion nanoparticles (UCNPs) and PS Ce6. As illustrated in Figure 6, the PLT system was prepared by adding UCNPs and Ce6 into polyacrylic acid-n-octylamine (PAAO) micelle and then coating it with PLTM. PLTs were separated by differential centrifugation and PLTM were extracted by repeated freezing and thawing. Then, the membrane protein was coated on the core of PAAO-UCNPS, and the final PMPAAO-UCNPs were prepared by ultrasound. In this system, the NPs were labeled with radionuclide ^125^I to guide the imaging of PDT for irradiating the plaque localization precisely, to avoid possible damage to the surrounding healthy tissue. PLTM coating contributes to the specific targeting of macrophage-derived foam cells, the markers and main components of early atherosclerotic plaques in the treatment system. Compared with the control group, H&E staining and SPECT/CT imaging showed that this PLT-like drug delivery system could accurately target atherosclerotic plaques *in vivo*. Antitumor study confirmed such PLT-mimicking system combined with PDT can significantly mitigate the progression of atherosclerosis in a mouse model.

### 3.4. Other natural cells

In addition of RBC, cancer cells and PLTM, there are many other types of cell membrane-bionic-based nanomaterials for phototherapy, including MDSCs, macrophage, plant cell membranes, and so on. These membranes can be used either alone or in combination for enhanced phototherapy.

#### 3.4.1. Myeloid-derived suppressor cells

The membranes of MDSCs are derived from bone marrow and also play an important role in immunosuppression such as tumor, angiogenesis, and metastasis (Talmadge & Gabrilovich, 2013; Kumar et al., 2016). They can be recruited into solid tumors and can be used for enhanced tumor targeting (Liu et al., 2018a,b; Zhang et al., 2020a,b). In recent years, researchers have combined MDSC-membrane with tumor phototherapy for anti-tumor.

**Figure 7.**
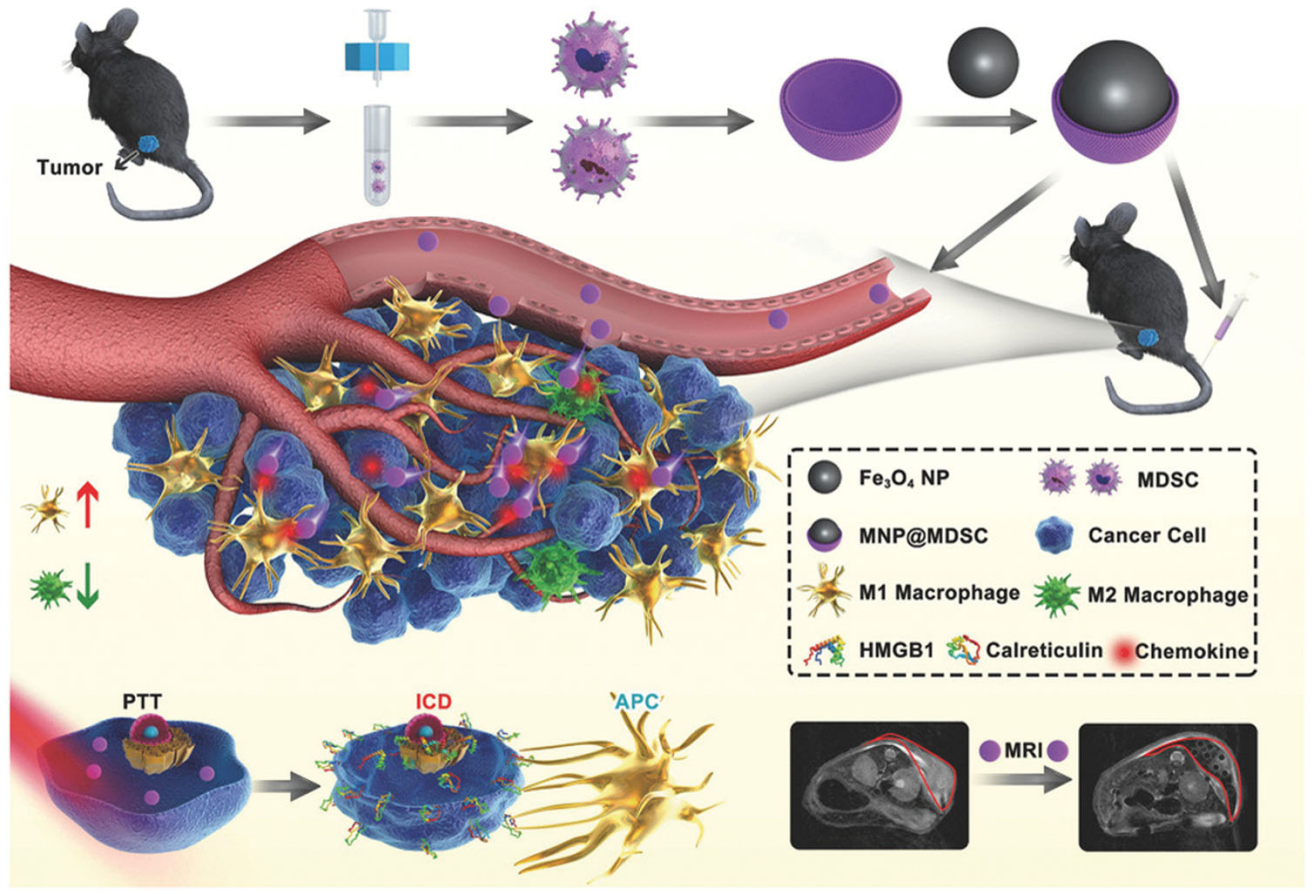
Schematic illustration of the synthesis of MNP@MDSC and its application in cancer theranostics. Copyright 2018, John Wiley and Sons (Yu et al., 2018).

Yu et al. (2018) designed a MDSC membrane-coated Fe_3_O_4_ NP (MNP@MDSC) for enhanced PTT (Figure 7). Results showed that when MNP@MDSC was incubated with macrophages of RAW264.7 mice, the ability of cell uptake was 5–10-fold lower than that of MNP, indicating that MNP@MDSC has the ability of immune escape. *In vivo* pharmacokinetic test exhibited the circulation time of MNP@MDSC treated group and MNP@RBC treated group was significantly longer than that of MNP treated group. Moreover, in this study, compared with the control group, the levels of high mobility group 1 protein (HMGB1) and Calreticulin were significantly increased in the MNP@MDSC group after PTT, indicating the occurrence of PTT-induced ICD. Beyond this, the synergistic effect of MNP@MDSC and PTT significantly increased the number of tumor-infiltrating CD8^+^T cells, reprogrammed tumor-related macrophages from M2 to M1, and decreased the metabolic activity of tumor cells, thus enhancing the anti-tumor response.

#### 3.4.2. Macrophage

Macrophage (M ø) is a kind of white blood cell. As important nonspecific immune cells, M ø contains a powerful lysosome system to treat foreign bodies in the body in a fixed or free state. For example, cell fragments, pathogens, and cancer cells for phagocytosis and digestion. It has been proved that macrophage membrane-modified NPs can achieve tumor-targeted chemotherapy in the form of controlled release under the stimulation of TME (Liu et al., 2020).

**Figure 8.**
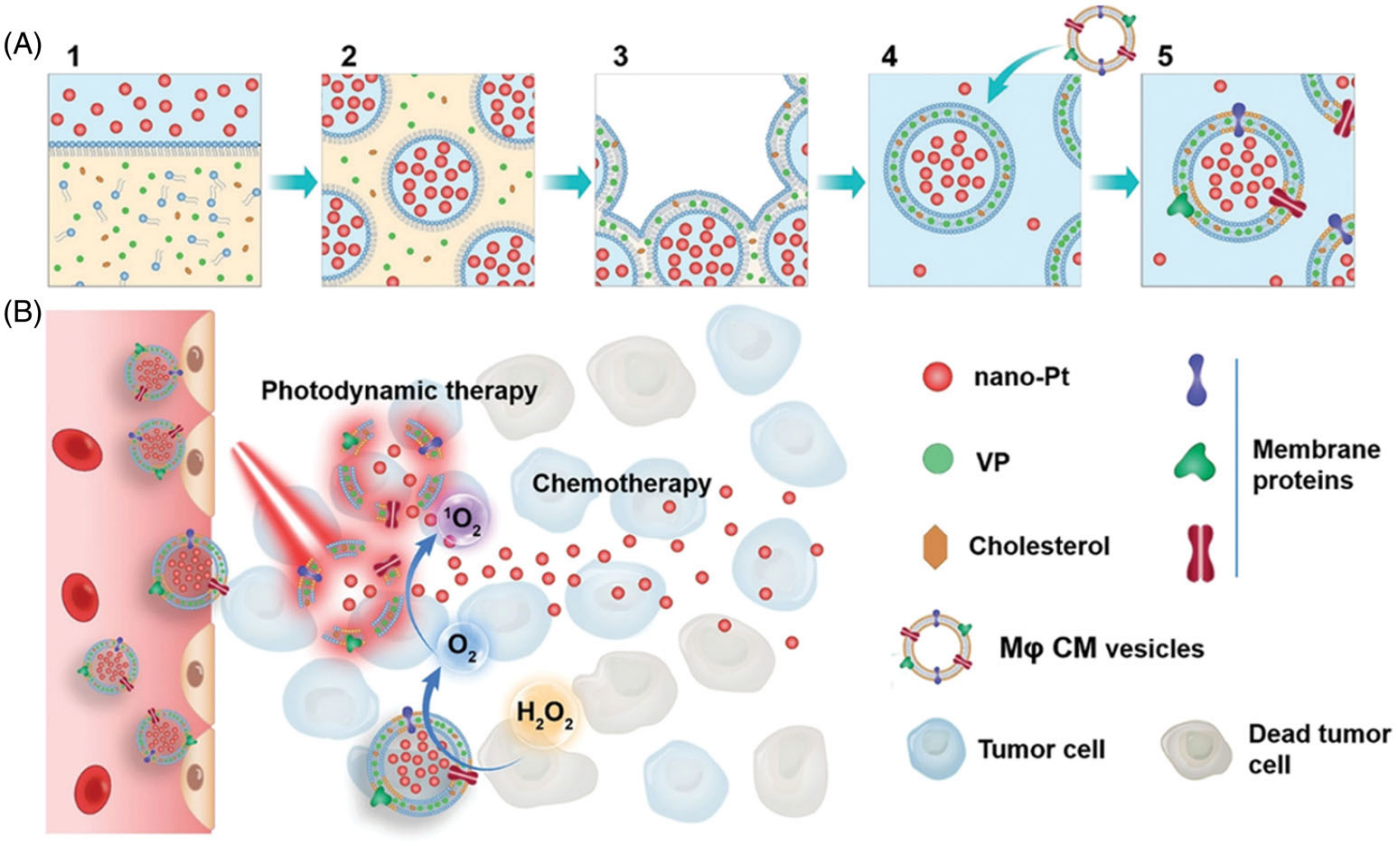
Schematic illustration of the fabrication (A) of nano-Pt/VP@MLipo and (B) chemophototherapy performance in tumors. Copyright 2018, Royal Society of Chemistry (Ouyang et al., 2018).

Compared with the traditional tumor-targeting therapy mediated by specific ligand-receptor binding, based on the inherent chemotaxis of macrophages and the ability of biosynthesis of tumor inhibitory factors, it shows great advantages in the accurate treatment of tumors. Therefore, macrophage membrane-based phototherapy has attracted attention for researchers. For instance, Liu et al. (2021) developed a macrophage membrane-coated liposome named ‘nanometer Pt/VP@MLipo’, by uploading Nano-Pt into liposomes by the reverse-phase evaporation, and the PS verteporfin (VP) into the lipid bilayer to endow PDT activity (Figure 8). Mouse macrophage membranes are hybridized into liposome membranes to endow them with biomimetic and targeting functions. Results showed that, oxygen catalyzed by platinum (Pt) NPs can enhance VP-mediated PDT at the tumor site. In turn, PDT triggers the ‘ultra-fast’ release of Pt NPs (light for 5 min, release > 95%) by enhancing the permeability of liposome membranes. Ultra-small Pt NPs can then penetrate into the tumor tissue. Interestingly, the catalytic production of O_2_ can also enhance the motility and tumor osmosis of Pt NPs, and enhance the effect of chemotherapy. *In vivo* results demonstrated that a single injection of biomimetic Pt NPs liposomes combined with light could effectively inhibit the growth of invasive 4T1 breast tumor (inhibition rate 90%) and lung metastasis (inhibition rate 100%), and prolong animal survival time (median survival time 72%).

#### 3.4.3. Plant cell membranes

Apart from animal cell membranes, it should be noted that plant cell membranes, such as powerful chloroplast thylakoids, H_2_O_2_ will gradually accumulate in chloroplasts under the influence of low temperature or high salt environment. In order to reduce the damage caused by high oxidative stress, plant leaves have evolved to form a powerful antioxidant system in the body. For example, the hydrogen peroxide decomposing enzyme on the thylakoid membrane can break down H_2_O_2_ into O_2_. Furthermore, the photosynthesis of green plants can release O_2_, and chlorophyll itself is a kind of fluorescent PS (Sewelam et al., 2014; Wang et al., 2017). Based on this inspiration, Ouyang et al. (2018) designed biomimetic plant thylakoids for PDT guided by fluorescence imaging of tumors. They firstly extracted the functional thylakoid cell membrane from spinach and then squeezed by the extruder successfully prepared the nanothylakoids (NTs), the membrane with a particle size of 50 nm. H_2_O_2_ decomposing enzyme can catalyze the decomposition of tumor endogenous H_2_O_2_ and effectively alleviate the problem of hypoxia. Under the irradiation of the near-infrared laser, the energy level transition of fluorescent dye chlorophyll occurs, which transfers the energy to O_2_ and then produces ^1^O_2_, which realizes the PDT guided by fluorescence imaging of tumor.

#### 3.4.4. Hybrid membrane

**Figure 9.**
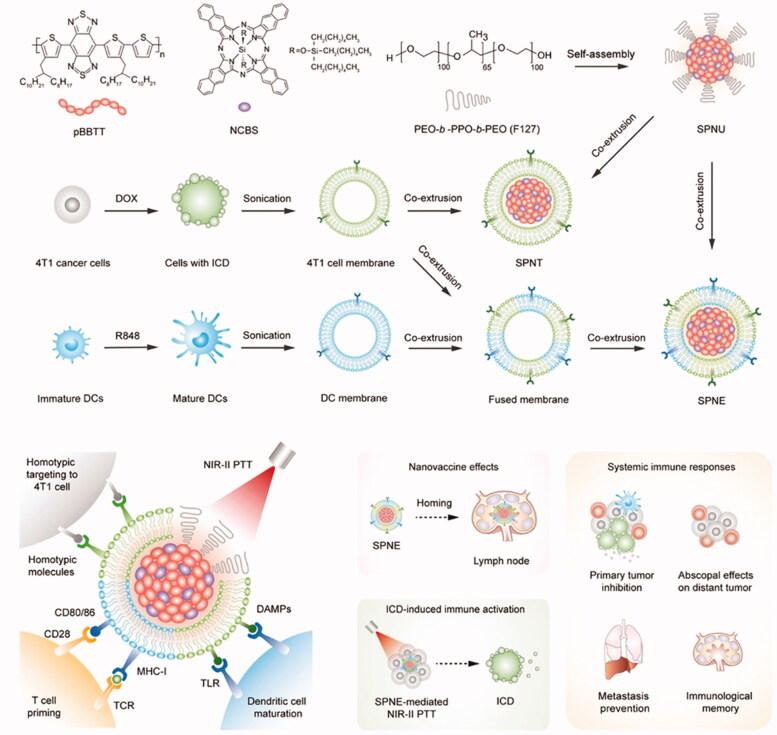
Schematic illustration of SPNE for synergistic NIR-II photothermal immunotherapy. Copyright 2021, John Wiley and Sons (Xu et al., 2021).

As different cell membranes have different characteristics, their functions can be integrated by fuzing two or more types of cell membranes into a hybrid membrane. Xu et al. (2021) reported a semiconducting polymer nanoengager (SPNE) for efficient NIR-II photothermal immunotherapy (Figure 9). SPNE uses NIR-II absorption polymer (semiconducting polymer nanoparticles [SPNs]) as photothermal core and uses fusion membrane from immune engineering tumor cells and DCs as cancer vaccine shell. On one hand, the homologous targeting of cancer cell membrane makes the SPNE highly accumulate in the tumor site, on the other hand, the existence of DC cell membrane endows SPNE with the function of interacting with T cells. In a word, SPNE can cross the biological barrier with the help of cancer cell membrane, and at the same time, the antigen presentation of DC cell membrane surface protein and T cells can promote T cell proliferation, recognize and kill tumor, and finally achieve the goal of photothermal synergism to enhance immunotherapy. The mice were injected intravenously, and the long-term anti-tumor immunity results showed that after 30 d of treatment, the proportion of T cells (CD45^+^CD3^+^CD44^+^) in lymph nodes of mice was about six times higher than that of tumor-bearing mice and healthy mice after 1 d of treatment. Moreover, central memory T cells (CD45^+^CD3^+^CD44^+^CD62L^+^) accounted for nearly 50% of the total T cells in mouse lymph nodes after 30 d, nearly five times higher than other groups, confirming the immune memory established by SPNE-mediated photothermal immunotherapy.

## 4. Biomimetic enzyme-based phototherapy

The so-called biomimetic enzyme is a kind of material that imitates the catalytic performance of biological enzyme. Gao et al. (2007) firstly discovered that Fe_3_O_4_ nanomaterials had peroxidase activity in 2007. Since then, as a kind of artificial enzyme that mimics the activity of natural enzyme, nano-enzyme (NE), together with natural enzyme, has received extensive attention in the diagnosis and treatment of cancer.

### 4.1. Metal biomimetic enzymes

In recent years, metal particles (such as Pt, gold [Au], etc.) show excellent biomimetic activity similar to catalase, peroxidase, peroxidase, and reductase (Gao et al., 2019; Ma et al., 2019; Zhang et al., 2020a,b; Xi et al., 2021). Therefore, researchers focused on developing novel types of metal biomimetic enzymes-based materials for enhanced phototherapy.

#### 4.1.1. Platinum (Pt)

**Figure 10.**
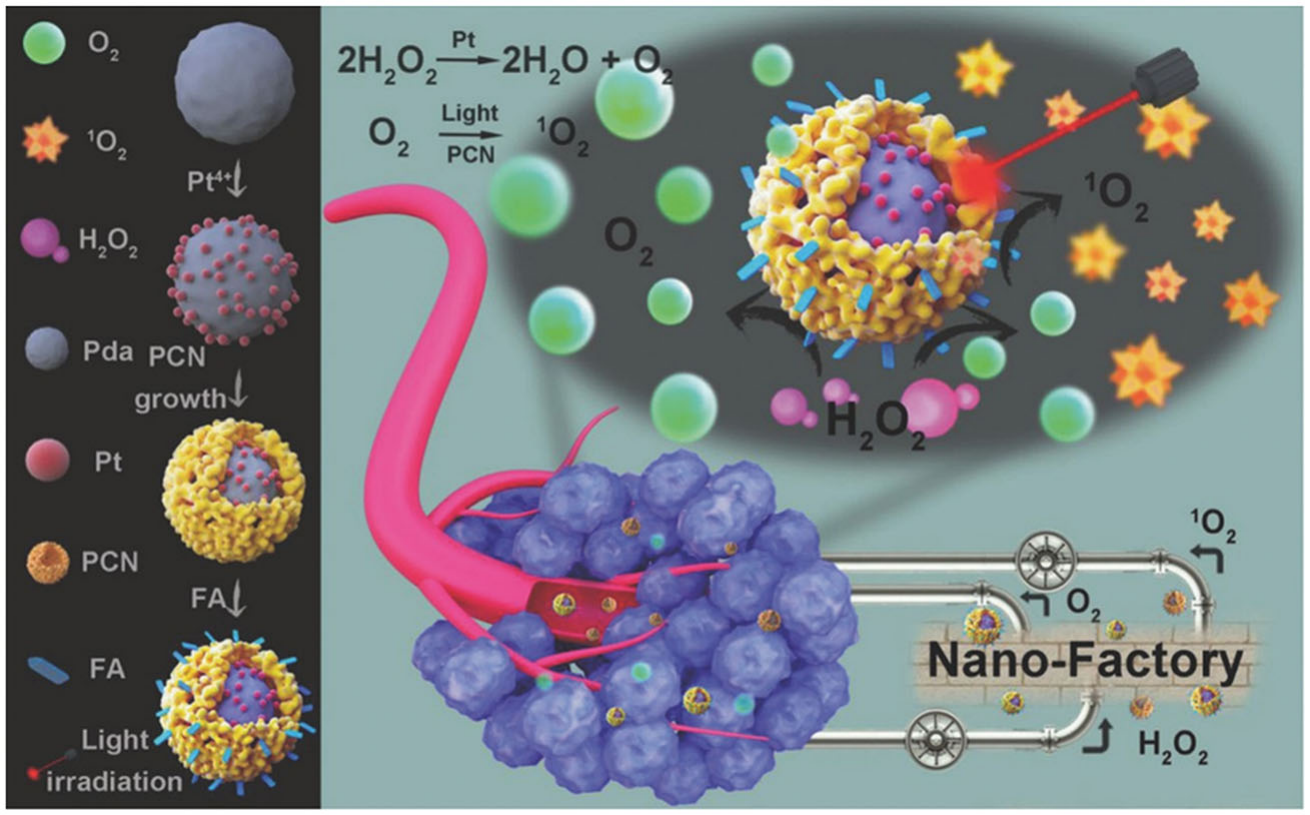
Schematic illustration of the core–shell nanofactory for enhanced tumor therapy. Copyright 2018, John Wiley and Sons (Wei et al., 2018).

In addition to the design of natural enzymes, some materials with enzyme-mimic activity have received increasing attention due to their unique advantages. For instance, Pt NPs can combine with other drugs and demonstrate excellent ability to catalyze H_2_O_2_. Based on this, researchers tried to develop novel types of Pt-based inorganic tumor phototherapy materials (Wei et al., 2018). One work worth mentioning is reported by Wang’ group (Wang et al., 2018). They produced a hybrid core-shell nanoplatform, with polydopamine as the core, Pt NPs interlayer, and zirconium porphyrin (PCN) as the shell. Pt NPs exhibit enzyme-mimic activity and can catalyze endogenous H_2_O_2_ to form O_2_. In the presence of light irradiation, O_2_ is then converted into ROS by zirconium porphyrin layer, thus enhancing the effectiveness of PDT (Figure 10). *In vitro* and *in vivo* studies have shown that the system can treat tumors more effectively by synergistically enhancing the regulation of PDT and TME. The study not only enriches the application of Pt-based nanomaterials in cancer treatment, but also provides guidance for the design of other nanosystems to treat cancer.

**Figure 11.**
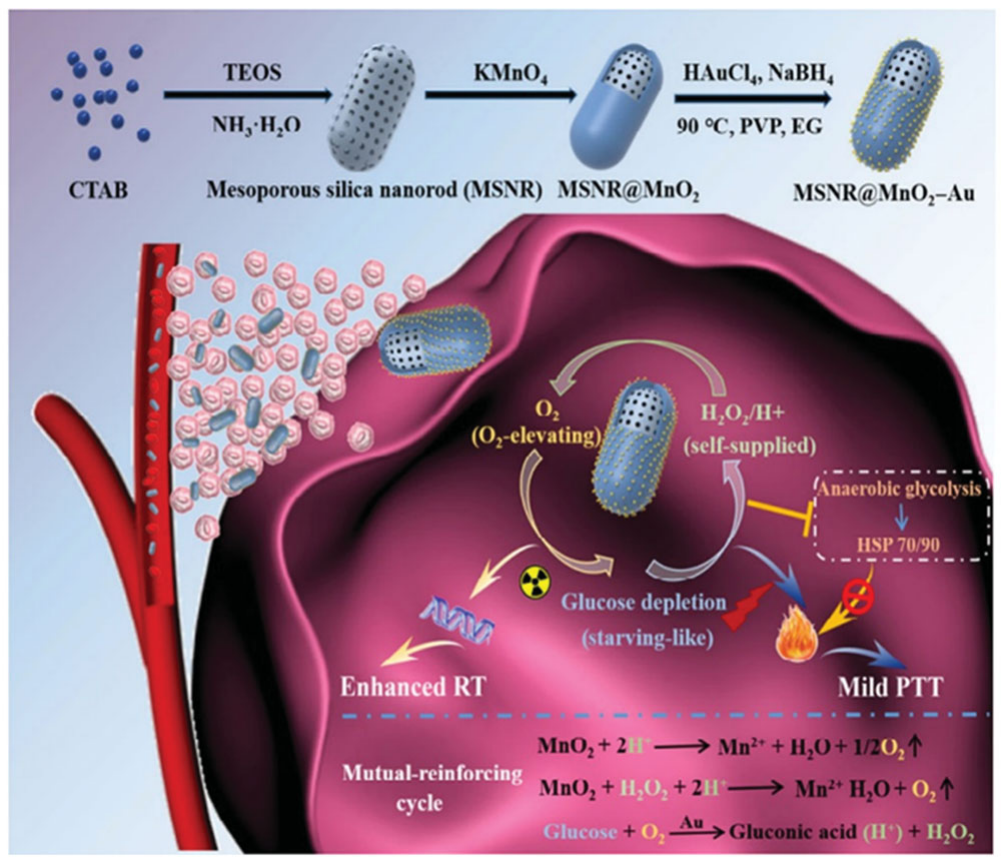
Schematic illustration of the MSNR@MnO_2_–Au-mediated mild PTT and enhanced RT in cancer cells. Copyright 2020, Springer Nature (Liu et al., 2019).

#### 4.1.2. Gold (Au)

Au can penetrate cells and cell compartments (less than 2 nm) and cause cell necrosis (Lou-Franco et al., 2021). Therefore, NEs with mimic peroxidase activity can catalyze the decomposition of hydrogen peroxide and produce ROS (Fan et al., 2018). In addition, Au NPs also have the activity of simulating glucose oxidase (GOX) (Gao et al., 2019; Liu et al., 2019). Therefore, the catalytic activity of Au can be applied for phototherapy.

In light of this situation, Yang et al. (2020a) designed and synthesized a rod-shape biomimetic composite inorganic nanoenzyme (MSNR@MnO_2_-Au) for the treatment of hypoxic tumors. First of all, the enzyme has the special shape of mesoporous silica nanorods and has excellent cell uptake ability. After entering the cell, in the hypoxic environment of the tumor, MnO_2_ could catalyze the decomposition of endogenous H_2_O_2_ to O_2_, thus further enhancing the efficacy of radiotherapy and improving the enzyme activity of AuNPs. Synchronously, Au NPs could catalyze the oxidation of glucose to gluconic acid and hydrogen peroxide, providing a large amount of H^+^/hydrogen peroxide, which can maximize the catalytic efficiency of MnO_2_ and further accelerate the formation of local O_2_. Moreover, down-regulate the expression of heat shock protein (HSP) induced by glucose consumption to achieve starvation treatment and mild PTT. Therefore, this mutually reinforcing cycle can achieve H^+^/H_2_O_2_ self-supply to accelerate O_2_ production and sustained glucose consumption, thereby alleviating tumor hypoxia and improving tumor thermal sensitivity, and ultimately enhance anti-tumor efficiency. The bio-mimic hybrid NE provides a new idea for nano-catalytic therapy of hypoxic tumors (Figure 11) .

### 4.2. Biomimetic nanoscale metal-organic framework

Burgeoning MOFs are a kind of immobilization of enzymes material. Their ordered porous structure provides rich and highly active metal sites, which makes MOFs a biomimetic catalytic material with a unique attraction.

**Figure 12.**
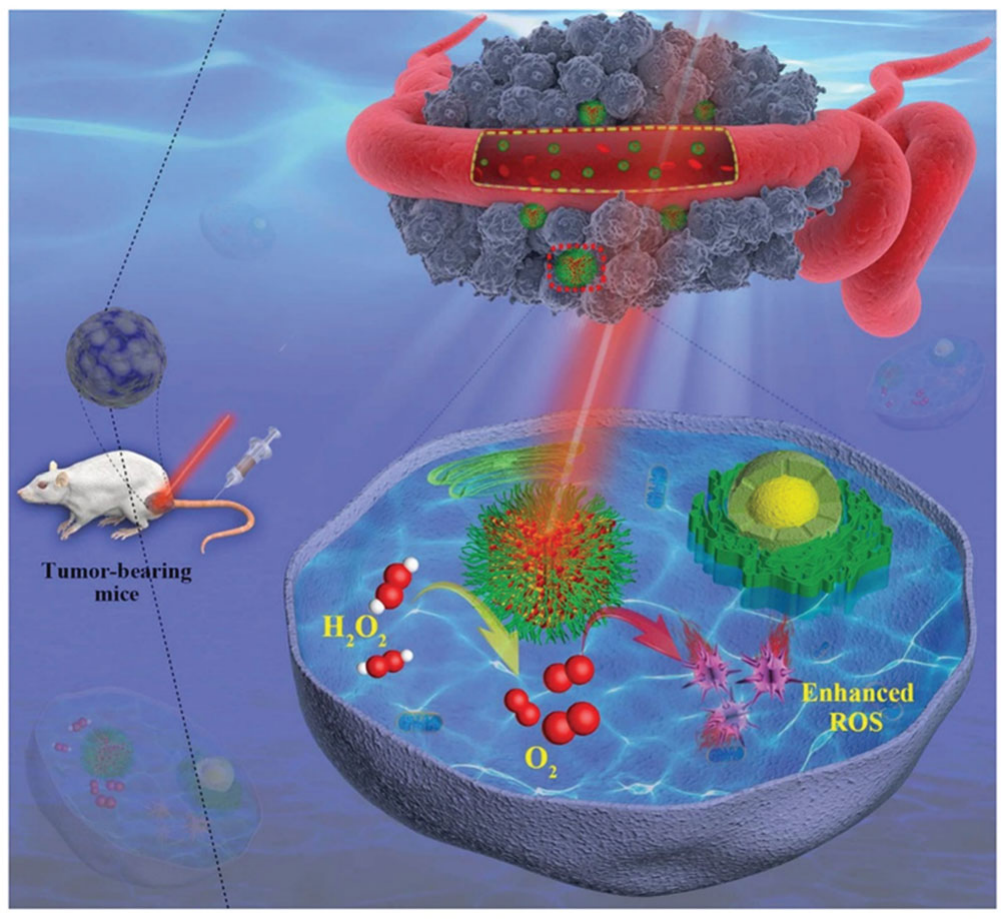
Schematic illustration of MOF-derived mesoporous NE for enhanced PDT of cancer. Copyright 2019, John Wiley and Sons (Wang et al., 2019a).

As illustrated in Figure 12, Wang et al. (2019a,b) extracted a multi-functional mesoporous NE, from metal-organic skeleton (MOFs) for in situ generations of endogenous O_2_ to improve the therapeutic efficiency of PDT. By using a one-step annealing strategy and biocompatible PDA and PEG modification, a multifunctional mesoporous MCOPP enzyme with MOFs as a precursor was successfully synthesized. The hypoxia of the TME was alleviated by catalytic reaction of NE and endogenous H_2_O_2_ to O_2_. In addition, NE loaded with Ce6 can be used as an oxygen donor to increase local O_2_ concentration and increase the production of ROS, thus significantly improve the therapeutic effect of anti-tumor PDT *in vitro* and *in vivo*. When co-incubated with MCOPP- Ce6, the death rate of 4T1 cells under hypoxia was lower than that of 4T1 cells under normoxia, and the expression of HIF-1 α protein was significantly down-regulated. *In vivo* experiment, compared with the control group, the tumor sections of MCOPP-Ce6 treated group showed a significant decrease in hypoxic immunofluorescence, confirming that MCOPP-Ce6 reduces the degree of tumor hypoxia by catalyzing the conversion of H_2_O_2_ to O_2_. H&E staining showed that the tumor tissue of MCOPP- Ce6 + light group was obviously damaged, which further verified the enhancement of PDT effect. Thus, in the presence of laser irradiation, MCOPP-Ce6 could completely suppress tumor growth with negligible side effects.

### 4.3 N-Doped carbon (NC) biomimetic nanomaterials

In 2018, Fan et al. (2018) confirmed that nitrogen-doped carbon (NC) nanomaterials possess high mimicking NE activities and can be used to regulate ROS. In addition, carbon nanomaterials can also absorb NIR light and have good biocompatibility, which makes them an ideal carrier material for cancer phototherapy.

**Figure 13.**
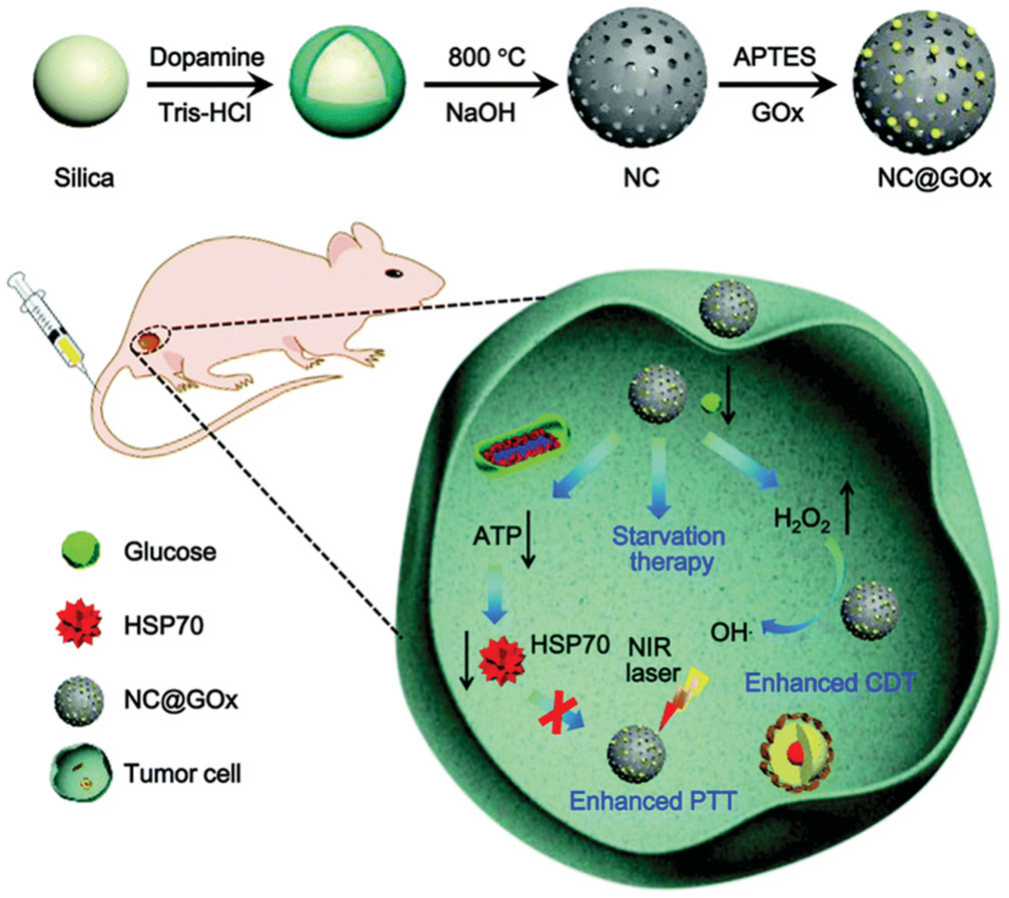
Scheme of NC@GOx NPs for combination therapy. Copyright 2020, RSC Pub (Xu et al., 2020).

Based on the advantages of NC nanomaterials, Xu et al. (2020) modified GOx on N-doped carbon NPs to construct a biomimetic nanoenzyme (NC@GOxNPs), which can enhance the anti-tumor effect of PTT and chemodynamic therapy (CDT) through starvation therapy (ST). As illustrated in Figure 13, for one aspect, not only can GOx break down glucose to cut off the tumor’s energy and nutrition to supply ST, but also it can reduce the level of ATP and down-regulate the level of HSP, which creates a more suitable microenvironment for improving the efficacy of PTT. For another aspect, the hydroxyl radical produced and ROS by H_2_O_2_ catalyzed by NC NPs further enhanced CDT. *In vitro* and *in vivo* experiments show that NC@GOxNPs can effectively kill cancer cells. Obviously, this study also provides a new strategy for the use of biomimetic NEs in the cooperative treatment of cancer.

### 4.4. Hybrid nanozyme

**Figure 14.**
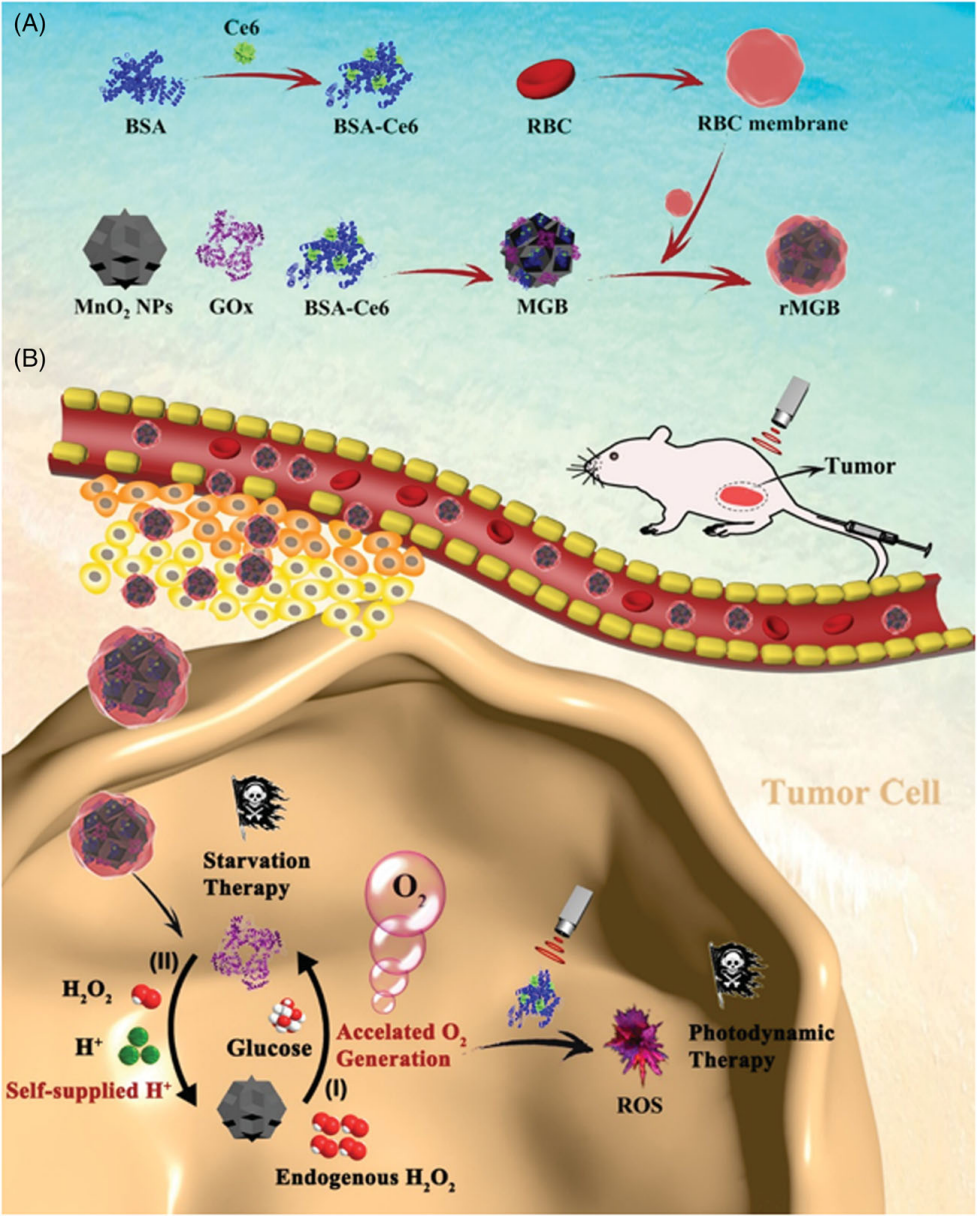
(A) Schematic design of the biomimetic hybrid nanozyme rMGB. (B) The scheme of the biomimetic hybrid nanozyme rMGB for alleviating tumor hypoxia and enhancing PDT and starvation therapy against hypoxic tumors. Copyright 2019, American Chemical Society (Yang et al., 2019).

Because different biomimetic enzymes have different characteristics, we can integrate their functions by synthesizing two different biomimetic enzymes to exert higher anti-tumor function. For example, Yang et al. (2019) designed a new biomimetic hybrid nanozyme (named rMGB) with high enzyme activity for hypoxic tumor therapy by using the strategy of mutual promotion between NE (MnO_2_) and natural enzyme (GOx) (Figure 14). In the tumor hypoxia environment, MnO_2_ in the rMGB biomimetic NE system can react with endogenous H_2_O_2_ to form O_2_, thus increasing the enzyme activity of GOx to accelerate the glucose consumption of tumor ST; meanwhile, GOx oxidizes glucose to produce gluconic acid, which provides a large amount of H^+^. These H^+^ can maximally improve the catalytic efficiency of manganese dioxide, further accelerate the production of local O_2_, reduce tumor hypoxia and improve the photodynamic effect. The effect of anticancer therapy in vivo showed that the rMGB/laser group combined with PDT, and ST had the best synergistic effect. This biomimetic hybrid NE is expected to be a potential oxygen donor for hypoxic tumor therapy.

For another example, Wang et al. (2020a,b) designed and fabricated a therapeutic nanoplatform Au_2_Pt-PEG-Ce6 with dual biomimetic activity. With the increase of the concentration of Au_2_Pt, the oxygen production rate increased gradually, which indicated that the NE had excellent catalase-like activity. Natural peroxidase (POD) substrate 3, 3′,5, 5′-Tetramethylbenzidine (TMB) was used to detect the POD-like activity of Au_2_Pt. At 652 nm, there is blue oxidized TMB (OxTMB) absorption, indicating that Au_2_Pt has POD-like activity and catalyzes H_2_O_2_ to form **^.^**OH. Therefore, Au_2_Pt has both catalase-like and POD-like activities, which can not only produce O_2_ to relieve tumor hypoxia and enhance PDT efficiency but also produce **^.^**OH for chemical dynamic therapy (CDT). More importantly, *in vitro* and *in vivo* data showed that Au_2_Pt can effectively inhibit tumor growth, and has few side effects on normal tissue. Under the combined irradiation of 650 and 808 nm, the survival rate of Hela cells incubated with Au_2_Pt-PEG-Ce6 decreased to 12%, which was significantly lower than that of 650 or 808 nm laser (55%) or 808 nm laser (34%). The results showed that the combination therapy could significantly improve the therapeutic effect, which was further confirmed by animal experiments.

## 5. Conclusion and perspectives

Phototherapy has shown extensive potential for cancer treatment. However, limitations still exist including but not limited to immune recognition, blood clearance, and low targeting. Prompted by nature, bionics is a feasible way to overcome these limitations through a unique biological interface. This review summarizes the recent progress of camouflage preparations for tumor phototherapy.

The application of cell membrane camouflage reagents in bionic phototherapy is the most common method. As a kind of natural membrane, the characteristics of the erythrocyte membrane save a lot of space for loading cargos. Moreover, due to the existence of its markers, it avoids the clearance of immunogenicity and prolongs the circulation time. This cell-membrane coating method does not affect the biological activity and photophysical properties of the original uncoated phototherapeutic agents, and can release the encapsulated drugs in a light-triggered manner for synergistic and combined therapy. Among the natural membranes, the RBC carrier has become one of the most popular way to delivery phototherapy agents. PLTM stands out because of its unique targeting ability. In the presence of glycoprotein (GP) Ib (GPIb), P-selectin, and CD44 receptors, PLTM camouflage reagents have specific adhesion of tumor tissues and absence of immunogenic clearance. The biofilm carrier derived from cancer cell membrane inherits the complex protein components on the membrane surface, so it retains the homologous targeting ability. Other cell membranes, including but not limited to MDSC membranes, macrophage membranes, fusion membranes, and plant cell membranes, endow membrane camouflaged carriers with functions, such as immune escape and tumor targeting. Enzyme is a widely used catalyst, and its synthetic potential has long been recognized. With its excellent biological activities similar to catalase, POD, and reductase, biomimetic enzyme can catalyze the production of ROS, hydroxyl radicals or heat to enhance the effect of phototherapy. In order to give full play to the potential of enzymes, it is usually necessary to redesign or optimize enzymes for specific applications. In recent years, many materials have been used as biomimetic NEs in tumor phototherapy, such as metal, MOF, NC, etc., because of their unique advantages.

Despite the advantages of biomimetic drug delivery systems, there still exist some challenges needed to be addressed. Biosafety is the primary consideration, especially for cancer cell membranes. Potential carcinogenicity is the greatest risk for cancer cell membrane-modified nanomaterials, so it is necessary to strictly remove genetic material from cancer cells to ensure safety. In addition, in order to develop multi-functional smart cell membrane coating nanomaterials, it is inevitable to modify the membrane, and some side effects may occur at the same time. To solve this problem, it is necessary to strictly analyze the toxicity of cell membrane coated nanomaterials and cell culture technology with stable production capacity. However, up to now, most studies have focused on acute toxicity, but ignore long-term toxicity, especially the pharmacokinetics behavior of cancer cell membrane and metal biomimetic enzyme materials to healthy organs. In addition to cancer cell membranes, the biosafety of immune cell membrane-based nanomaterials is also crucial for phototherapy. Compared with other cells, immune cells have specific tumor immune recognition ability, for example, T cell receptor (TCR), an immune recognition protein on T cell membrane, can selectively recognize tumor surface antigen-activated T cells, thus showing high affinity and killing ability to tumor. Due to the high mutagenicity of tumor cells and the influence of immune microenvironment, tumors usually lead to ‘immune escape’. The dual targeting strategy may provide a more reliable and effective method to improve the tumor-targeted therapeutic effect of nano-drugs.

Another challenge is the preparation method of cell membranes. There are some limitations of traditional cell membrane separation methods, such as the huge sample demand, low yield, tedious operation steps, and time-consuming problems, thus leading to the low membrane encapsulation efficiency. Therefore, when coating NPs on the cell membrane, professional equipment and methods, such as microfluidic technology, is needed to improve the efficiency and coverage of the coating, so as to ensure the orientation of functional proteins on the cell membrane. After preparation, it is necessary to characterize the cell membrane-coated NPs, including their physicochemical and biological properties, in order to confirm that the cell membrane has been successfully coated on the surface of the NPs.

Last but not least, the role of metal biomimetic enzymes including MOF biomimetic enzyme composites should be further studied, and more variety of biomimetic catalysis enzymes except POD should be found to expand their applications. At present, a large number of studies are focused on erythrocyte membrane coated NPs, while other cell membrane technologies still invasive basic research, which requires more attention.
